# Comparative metagenomics reveals impact of contaminants on groundwater microbiomes

**DOI:** 10.3389/fmicb.2015.01205

**Published:** 2015-10-31

**Authors:** Christopher L. Hemme, Qichao Tu, Zhou Shi, Yujia Qin, Weimin Gao, Ye Deng, Joy D. Van Nostrand, Liyou Wu, Zhili He, Patrick S. G. Chain, Susannah G. Tringe, Matthew W. Fields, Edward M. Rubin, James M. Tiedje, Terry C. Hazen, Adam P. Arkin, Jizhong Zhou

**Affiliations:** ^1^Institute for Environmental Genomics, Department of Microbiology and Plant Biology, University of Oklahoma, NormanOK, USA; ^2^The Biodesign Institute, Arizona State University, TempeAZ, USA; ^3^CAS Key Laboratory of Environmental Biotechnology, Research Center for Eco-Environmental Sciences, Chinese Academy of SciencesBeijing, China; ^4^Bioscience Division, Los Alamos National Laboratory, Los AlamosNM, USA; ^5^United States Department of Energy, Joint Genome Institute, Walnut CreekCA, USA; ^6^Department of Microbiology, Montana State University, BozemanMT, USA; ^7^Center for Microbial Ecology, Michigan State University, East LansingMI, USA; ^8^Department of Civil and Environmental Engineering, University of Tennessee-Knoxville, KnoxvilleTN, USA; ^9^Department of Earth and Planetary Sciences, University of Tennessee-Knoxville, KnoxvilleTN, USA; ^10^Department of Microbiology, University of Tennessee-Knoxville, KnoxvilleTN, USA; ^11^Biosciences Division, Oak Ridge National Laboratory, Oak RidgeTN, USA; ^12^Department of Bioengineering, Lawrence Berkeley National Laboratory, BerkeleyCA, USA; ^13^Earth Sciences Division, Lawrence Berkeley National Laboratory, BerkeleyCA, USA; ^14^State Key Joint Laboratory of Environment Simulation and Pollution Control, School of Environment, Tsinghua UniversityBeijing, China

**Keywords:** metagenomics, bioremediation, groundwater microbiology

## Abstract

To understand patterns of geochemical cycling in pristine versus contaminated groundwater ecosystems, pristine shallow groundwater (FW301) and contaminated groundwater (FW106) samples from the Oak Ridge Integrated Field Research Center (OR-IFRC) were sequenced and compared to each other to determine phylogenetic and metabolic difference between the communities. *Proteobacteria* (e.g., *Burkholderia*, *Pseudomonas*) are the most abundant lineages in the pristine community, though a significant proportion ( >55%) of the community is composed of poorly characterized low abundance (individually <1%) lineages. The phylogenetic diversity of the pristine community contributed to a broader diversity of metabolic networks than the contaminated community. In addition, the pristine community encodes redundant and mostly complete geochemical cycles distributed over multiple lineages and appears capable of a wide range of metabolic activities. In contrast, many geochemical cycles in the contaminated community appear truncated or minimized due to decreased biodiversity and dominance by *Rhodanobacter* populations capable of surviving the combination of stresses at the site. These results indicate that the pristine site contains more robust and encodes more functional redundancy than the stressed community, which contributes to more efficient nutrient cycling and adaptability than the stressed community.

## Introduction

About one third of global freshwater reserves are located in subsurface streams and aquifers and represent a critical source of fresh water for human consumption and irrigation (Gleick, 2000). The suitability of groundwater for human use is affected by the chemical properties of the groundwater and some reserves may naturally be unsuitable for human use without extensive purification (Foster and Chilton, 2003; Wada et al., 2014). In addition, significant portions of natural groundwater reserves are rendered unusable due to anthropogenic contaminations that results from by-products of industry, agricultural runoff, and human/animal waste (Kunin et al., 2008). Environmental contaminants not only affect the quality of groundwater directly but also alter various biogeochemical cycling processes by altering native microbial communities. The loss of biodiversity in groundwater communities as a result of contamination greatly impacts geochemical cycling within the groundwater ecosystem (Cardenas et al., 2008; Moreels et al., 2008). Thus, understanding the nature of stress response and geochemical cycling in contaminated groundwater communities is critical for the design of effective *in situ* restoration strategies to rehabilitate and protect groundwater.

Metagenomic sequencing of community DNA from pristine and contaminated environments provides a powerful approach for evaluating the effects of contamination on pristine ecosystems. To date, numerous metagenomic studies have been conducted on soil, freshwater sediments and surface waters (Tyson et al., 2004; Tringe et al., 2005; DeAngelis et al., 2010; Brisson et al., 2012; Martinez-Garcia et al., 2012; Inskeep et al., 2013). These data sets have been used for biogeographical comparisons of communities from similar environments (Kunin et al., 2008; Inskeep et al., 2013), to analyze geochemical cycling (Hemme et al., 2010; Inskeep et al., 2013), and for comparing microbial communities from drastically different environments (Tringe et al., 2005). While some contaminated groundwater systems have also been sampled (Hemme et al., 2010; Smith et al., 2012), few comparisons of geographically similar contaminated and pristine shallow groundwater samples have been conducted to date. These previous groundwater studies demonstrate that anthropogenic contamination heavily impacts community structure and function of groundwater communities, but the lack of proper controls limits a full understanding of the effects of contamination on biodiversity and metabolic potential in these systems.

To serve as a control for present and future metagenomic experiments involving contaminated groundwater, the metagenome of a pristine shallow groundwater system (well FW301) from the U. S. Department of Energy’s Integrated Field Research Center (OR-IFRC) at Oak Ridge, TN was sequenced. The pristine site lies in the West Bear Creek Valley approximately 2 km from the former S3 waste disposal ponds and occurs along the same geological strike as the contaminated research areas, including the FW106 site characterized previously (Hemme et al., 2010)^1^ The groundwater in the area is predominantly anaerobic and circumneutral (Schreiber et al., 1999), with periodic exposure to oxygen resulting from percolation of aerobic rainwater and up- and down-welling with local surface water sources. The contaminated FW106 site lies at the base of the contaminant plume emanating from the former S3 waste disposal ponds and as such is chronically exposed to high concentrations of nitric acid, uranium and other radionuclides (e.g., technetium), heavy metals and organic solvents. The objectives of this study were to pursue the following questions: (i) What is the phylogenetic and functional diversity of pristine and how does it compare to the stressed groundwater community? (ii) How robust is the pristine community to environmental perturbation? (iii) How does a microbial community adapt to severe environmental changes such as heavy metal contamination? To address the above questions, the pristine metagenome was characterized and compared to the re-sequenced contaminated metagenome of the contaminated groundwater microbial community. The results suggest that the pristine groundwater community is highly diverse and encodes a high degree of metabolic potential (e.g., metal resistance genes) and functional redundancy. Introduction of contamination not only reduces phylogenic diversity but also reduces metabolic diversity and redundancy, leading to truncated geochemical cycles.

## Materials and Methods

### Metagenomic Sampling and DNA Extraction

All samples were obtained from shallow groundwater located at the Integrated Field Research Center (OR-IFRC) at the Y-12 Federal Security Complex in Oak Ridge, TN. Well FW301 (Background site, Lat. 35.94 Long. -84.33) is located ∼2 km from Well FW106 (Area 3, adjacent to the former S-3 disposal ponds) with both wells located on the same geological strike along the Bear Creek Valley. The Background site represents an uncontaminated region designated as a baseline environment for comparisons to contaminated areas. The groundwater at the site is circumneutral (∼pH 7.0) and anaerobic but may be periodically exposed to oxygen resulting from percolation of aerated rainwater into the aquifer or from up- and down-welling with surface waters. Biomass and metagenomic DNA was isolated as previously described for the FW106 sample (Hemme et al., 2010). To summarize, following purging of several well volumes of water, a total of 500 L of groundwater were extracted from well FW301 (1/16/07 and 2/5/07) at a depth of 21 m by peristaltic pumps and biomass was collected by filtering with 0.2 μm Supor filters (Pall Corporation, Port Washington, NY, USA). High molecular weight community DNA was extracted using grinding, freezing–thawing, SDS-based methods and the purified DNA was treated with RNase (Zhou et al., 1996). Geochemical data (e.g., dissolved O_2_, metal concentrations, etc.) for the sites was independently collected and retrieved from the FRC web site for the time points closest to the groundwater extraction dates^2^ (**Supplementary Table S1**).

### Metagenomics Sequencing and Assembly

Sanger sequencing, assembly and annotation of the original FW106 metagenome was conducted as previously described at the JGI Production Genomics Facility (Hemme et al., 2010) and a similar method was used for Sanger sequencing of the FW301 metagenome. For FW301 Sanger sequencing, ∼109 Mb small insert (3 kb) pUC library was generated with the longest scaffold of length 4168 bp. Additional sequencing was conducted at Los Alamos National Laboratory using Illumina GAIIx PE and HiSeq SE machines. A total of 6,020 (FW106) and 18,995 (FW301) Mb were obtained, respectively, from all sequencing methods, with reads ranging from 36 to 150 bp depending on the sequencing method employed (**Supplementary Table S2A**). The original Sanger reads were quality trimmed (*q* = 20, window size = 50) and split into 100 bp fragments w/50 bp overlap. These reads were combined with quality trimmed Illumina reads (*Q*-score ≥ 2 and sequence length > 1/2 read length) and assembled using SOAPdenovo using 19–31 bp kmer’s^3^ Multi-kmer contigs were dereplicated and length-filtered to 150 bp. The combined datasets were then sequentially combined using newbler (<= 1000 bp; Chaisson and Pevzner, 2008) and minimus2 (>1000 bp and newbler output)^4^ The resulting contigs were extended and scaffolded by SSPACE using Illumina read information (**Supplementary Table S2B**), (Boetzer et al., 2011).

### Comparative Metagenomics Analysis

Final assemblies were uploaded to the Joint Genome Institute Integrated Microbial Genomes, Metagenomics Expert Review (IMG/mer) website (Markowitz et al., 2008, 2009, 2010, 2012) and MG-RAST (Meyer et al., 2008) for annotation and analysis (**Supplementary Table S2C**). A total of 119082 and 626833 protein-coding genes were identified in FW106 and FW301, respectively, using the IMG annotation (**Supplementary Table S2C**). Of these, 74229 (61.96%) and 345758 (54.91%) were assigned to clusters of orthologous genes (COGs) for FW106 and FW301, respectively (**Supplementary Table S2C**).

### Sequencing, Quality Filtering and Taxonomic Assignment of 16S Amplicons

16S rRNA genes were sequenced from the FW106 and FW301 metagenomes at the University of Oklahoma using an Illumina MiSeq machine based on the method described in Caporaso et al. (2012) and adapted from Caporaso et al. (2011). To summarize, the V4 region of the 16S rRNA gene was amplified using region-specific primers including Illumina flowcell adapter sequences. The reverse amplification primer contained a 12 bp barcode sequence allowing for pooling of up to 1500 multiple samples in each run. Following cluster formation on the MiSeq instrument, the amplicons were sequenced using primers complimentary to the V4 region and designed for paired-ends sequencing. A third sequencing primer in additional cycles was used for reading the barcodes. To support paired ends sequencing on MiSeq, the amplification primers were adapted from Caporaso et al. (2011) to include nine extra bases in the adapter region of both forward and reverse amplification primers and a pad region to avoid primer-dimer formation. The MiSeq preparation and sequencing protocol is described in Caporaso et al. (2012).

After assigning each sequence to its sample according to its tag/barcode, allowing for 1 to 2 mismatches, a total of 22,693 reads of 150 bp from both ends were obtained for the two samples (FW301: 9661, FW106: 13,032). These sequences were then trimmed based on quality scores using Btrim (Kong, 2011), and pair-end reads are merged into longer reads by FLASH (Magoč and Salzberg, 2011). Unqualified sequences were removed if they were too short or contained ambiguous residues. Chimeric sequences were discarded based on prediction by Uchime (Edgar et al., 2011). 18,551 sequences remained for further analysis. OTUs were clustered using RDP’s mcClust program (complete lineage clustering, unpublished results) at the 97% similarity level. A final total of 3091 OTUs were generated, and taxonomic annotations were assigned to each OTU’s representative sequence by RDB’s Naïve Bayesian 16S Classifier (Wang et al., 2007; Claesson et al., 2009). In summary, the classifier compares a sequence to a random subset of a data base consisting of all possible eight-base subsequences (words). Each sequence is classified 100 times and the results are used to create a joint probability distribution to determine the confidence level of assignment. Analysis has shown that sequences can be accurately assigned at the level of genus using a 50% confidence cutoff (Claesson et al., 2009).

To complement the MiSeq 16S sequencing, 16S fragments were extracted from the metagenomic read libraries for direct comparison to the MiSeq results. Reference 16S sequences were obtained from the Ribosomal Database and compared to the read libraries by Blastn. Best hits were extracted using *ad hoc* Perl scripts and the resulting sequence sets were assigned to taxa using the same method as the MiSeq results (described above). Because metagenomic reads do not necessarily correspond to the V4 region sequenced via the MiSeq protocol, common OTU’s could not be defined between the two datasets. In order to compare the distribution of sequences between the datasets, the OTU data for the various datasets was converted to abundance profiles indicating the frequency of specific genera within the datasets. The resulting genus-based abundance profiles were then tested for correspondence in R using Pearson’s method. To test for correlation at the OTU level, 16S reads obtained from the read libraries were combined with the MiSeq amplicons and clustered using CD-HIT with a similarity cutoff of 0.97 to define common abundance groups (Fu et al., 2012). The abundance of each cluster in each sample was calculated and the resulting abundance profiles were tested for correspondence in R using Pearson’s method.

**FIGURE 1:**
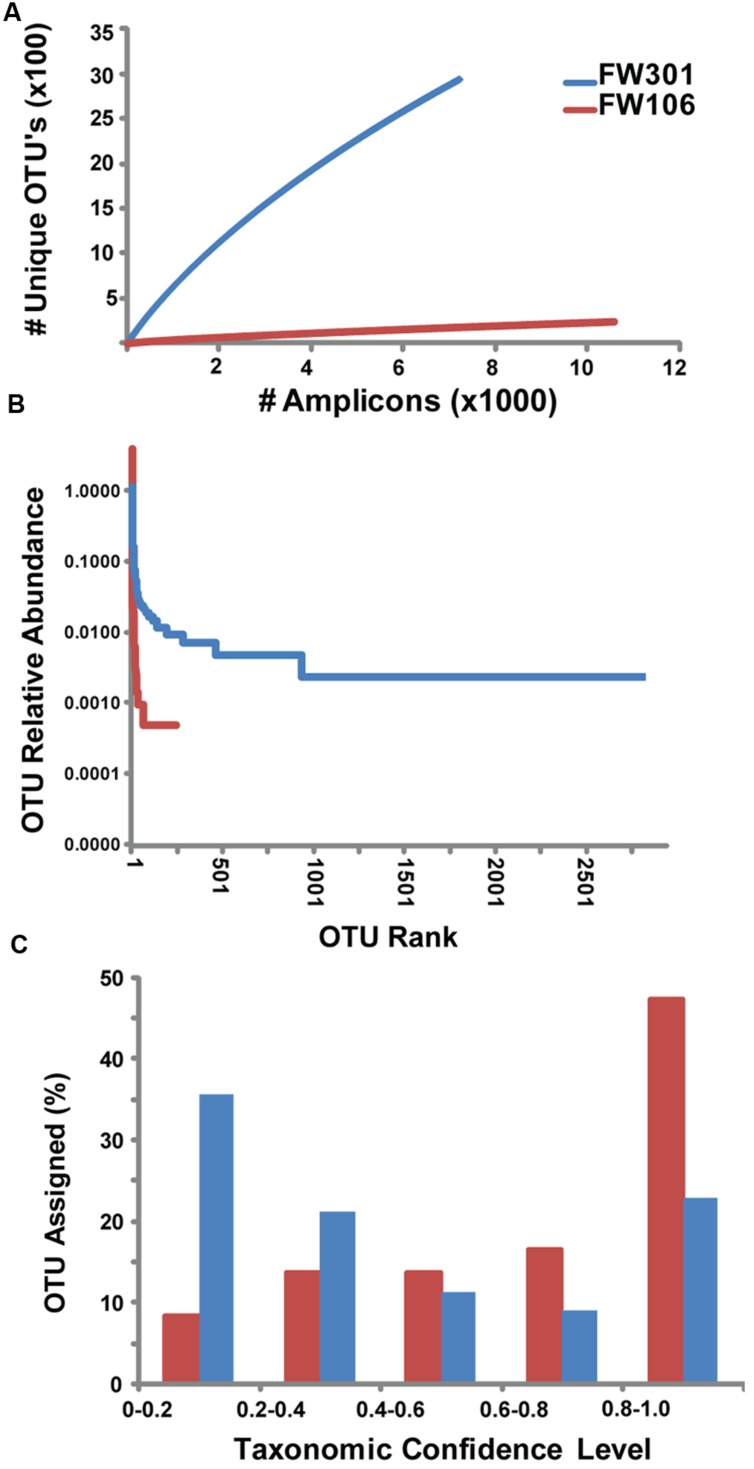
**Abundance and distribution of 16S amplicons within OTU’s for OR-IFRC communities.** **(A)** Rarefaction curve of # 16S amplicons based on unique OTU’s derived from MiSeq 16S amplicon data. **(B)** Rank abundance plot of relative abundance of 16S amplicons within OTU’s ranked by size (1 = largest OTU). Sequences were binned based on OTU population (e.g., for FW106, 1 OTU contained 8383 sequences, 1 OTU contained 507 sequences, etc.). The resulting sequence bins were ranked by abundance (1 = largest sequence bin). **(C)** Histogram of confidence levels of taxonomic assignments for 16S amplicons. Confidence level >0.5 (i.e., sequence assigned to taxon >50% of the time) is considered to be a valid taxonomic assignment.

**FIGURE 2:**
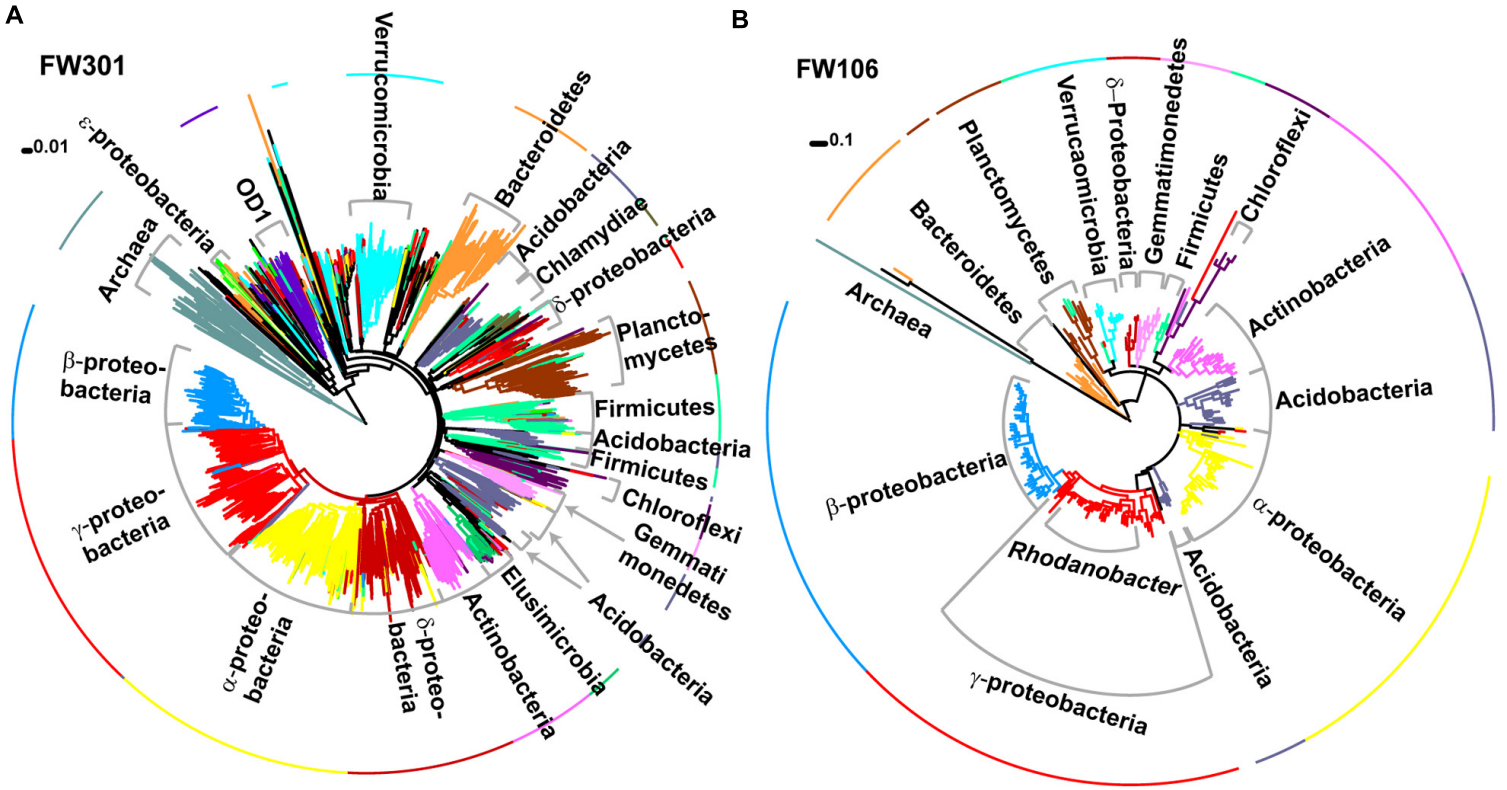
**Phylogenetic trees of 16S amplicons sequenced by MiSeq for **(A)** FW301 and **(B)** FW106.** Clades and the first ring are colored by phylogeny and labeled. Trees were generated in Mega 5.1 using neighbor joining **(A)** or maximum likelihood **(B)** methods. Note: a maximum likelihood tree for FW301 could not be resolved despite multiple attempts.

**FIGURE 3:**
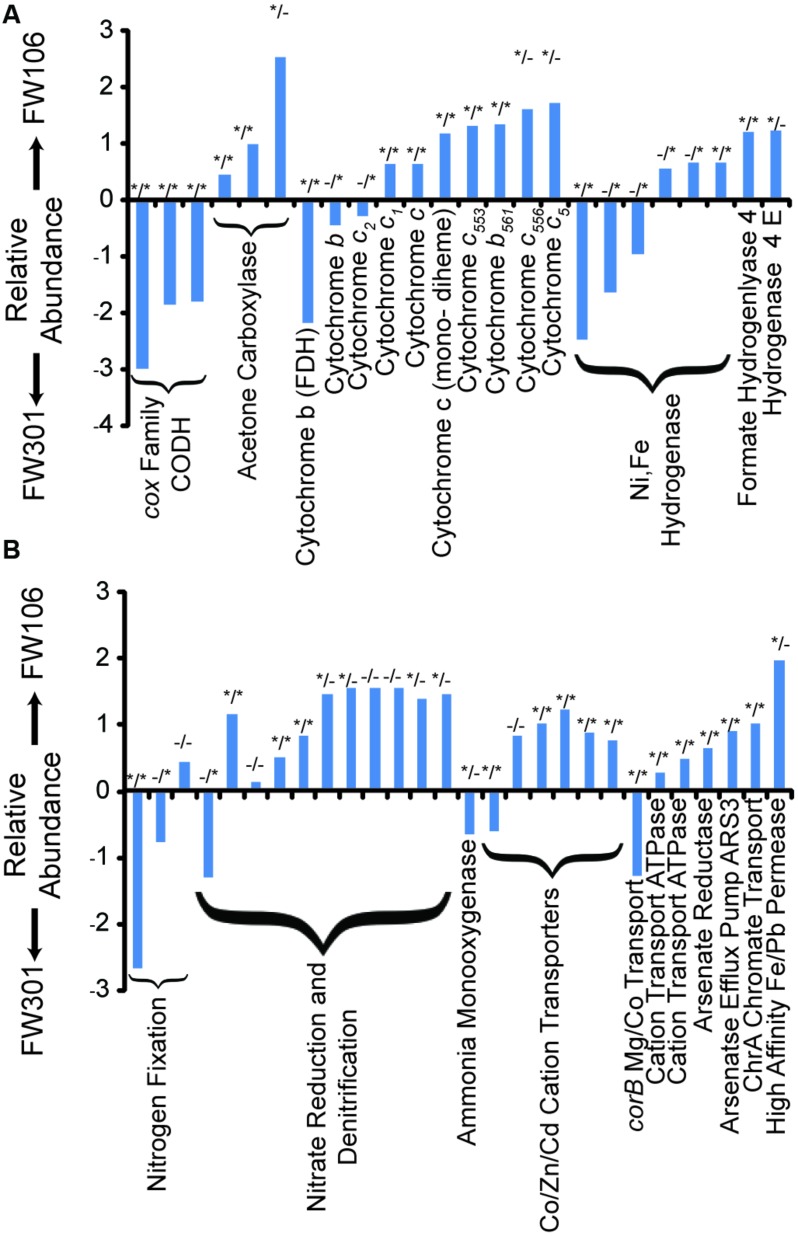
**Odds Ratios of OR-IFRC assigned to COGs.** Odds ratios were calculated as described in section “Materials and Methods” and plotted as ln odds ratio. Plots are divided into two sets of data for clarity: **(A)** carbon monoxide dehydrogenase (CODH), acetone carboxylase, cytochromes, and hydrogenases; **(B)** nitrogen metabolism and transport, and metal metabolism and transport. Positive values indicate overabundance of the COG in FW106, negative values indicate overabundance in FW301. Statistical deviation from unity for each data point (*p* value < 0.05) is indicated by asterisks by either χ^2^ test (left asterisk) or two-tailed Fisher’s exact test (right asterisk).

**FIGURE 4:**
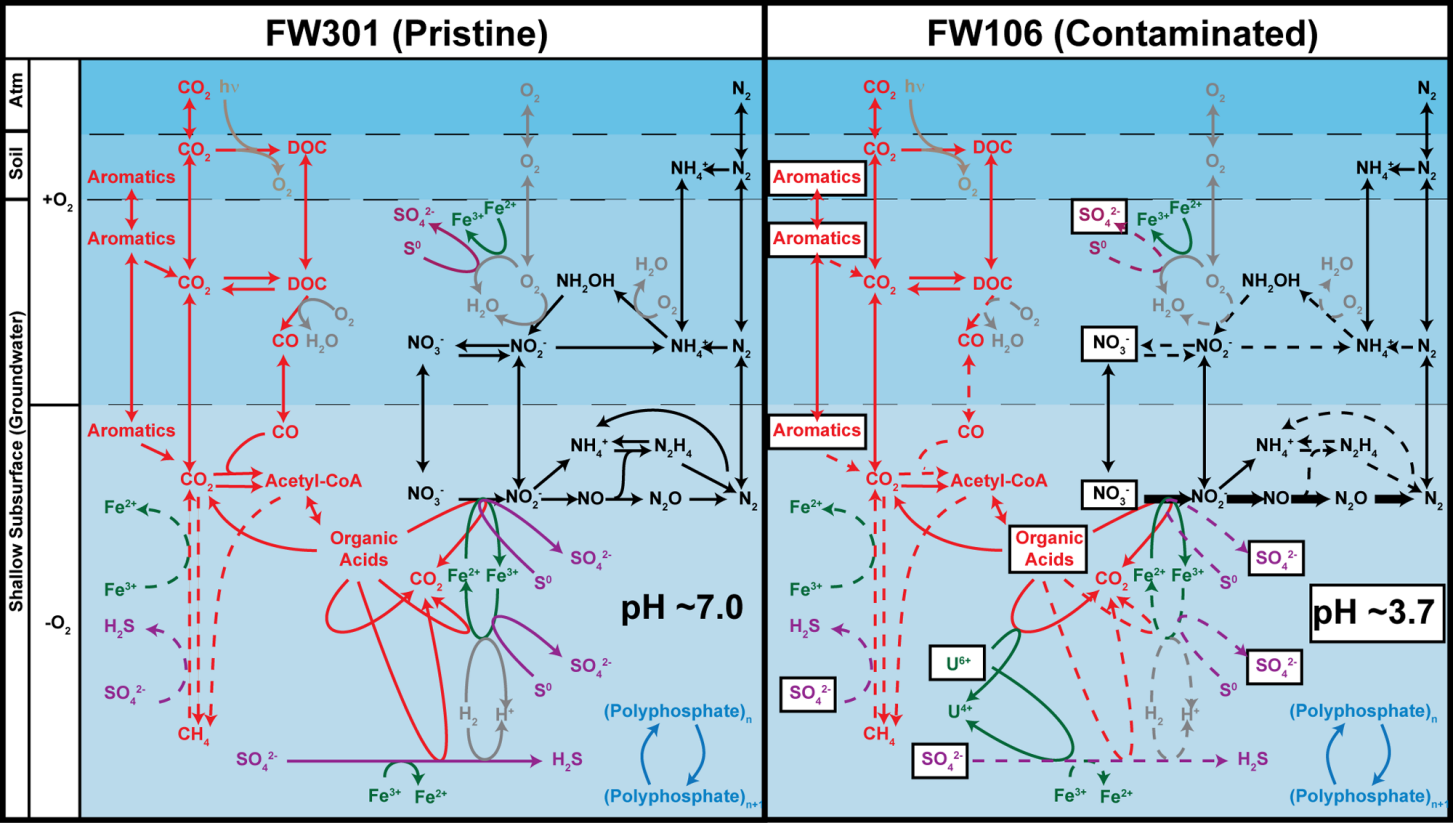
**Predicted geochemical cycling in the FRC subsurface based on metabolic profiles of pristine (FW301, **left)** and contaminated (FW106, **right)**.** The denitrification pathway genes are labeled in bold in the FW106 figure to indicate their overabundance in this metagenome compared to FW301. Dotted lines indicate either that the pathway genes were not detected in the metagenomes, detected in very low abundance, or that bacteria known to implement these pathways were not detected or in extremely low abundance (<1%). Contaminants present in high concentrations at the FW106 site are shown in white boxes. Partitions indicate different geochemical and electrochemical environments that may transiently exist at the OR-IFRC sites and do not necessarily represent the specific environmental partitioning present in these environments.

Plots in **Figures 1** and **3** were generated in Microsoft Excel and modified in Adobe Illustrator to improve fonts and increase legibility. The underlying data was not modified. **Figure 2** was generated in iToL and modified in Adobe Illustrator to add labels. The underlying data was not modified. **Figure 4** was created entirely in Adobe Illustrator.

Worked conducted at University of Oklahoma (Illumina sequencing and analysis), Joint Genome Institute (genome sequencing) and Oak Ridge National Laboratory (biomass isolation).

## Results

### Characteristics, Sequencing and Annotation of Metagenomes from Pristine and Contaminated Groundwater

The pristine groundwater was circumneutral (pH ∼7), in contrast to the contaminated site (pH ∼3.7; **Supplementary Table S1**). Past experimental analyses have shown that the contaminants present at the contaminated (e.g., nitrate, sulfate, organics, heavy metals) site were much higher than the ambient concentrations at the pristine site (Shelobolina et al., 2003; Moreels et al., 2008; **Supplementary Table S1**). Groundwater at both sites tends to show low concentrations of dissolved oxygen, suggesting the groundwater environments are typically anoxic (**Supplementary Table S1**). However, the communities are likely to be periodically exposed to oxygen due to up- and down-welling of surface waters and percolation of aerobic rainwater into the system. The background and contaminated areas lie along the same geological strike and are underlain by the same geology, mineralogy and structure (https://public.ornl.gov/orifc/FRC-conceptual-model.pdf; Watson et al., 2004; Kim et al., 2009). As such, it is assumed that FW301 and FW106 would show the same overall geochemical profiles in the absence of contamination. However, how this reflects on the microbial scale in terms of local geochemical variation and available microenvironments is unknown.

The pristine metagenome was sequenced using a combination of Sanger, Illumina GAIIx and HiSeq (**Supplementary Table S2**). A total of ∼15 and 60 Mb Sanger sequencing reads were obtained for the pristine and contaminated metagenomes (**Supplementary Table S2A**), respectively. Also, ∼183 and ∼104 Gb short read sequences were generated with the Illumina sequencing platforms (**Supplementary Table S2A**). The resulting sequences were assembled and ∼226 and ∼59 Mb assembled sequences were obtained for the pristine and contaminated metagenomes, respectively (**Supplementary Table S2B**). The maximum scaffold lengths were ∼80 and ∼280 kb for the pristine and contaminated metagenomes, respectively (**Supplementary Table S2B**). Also, IMG annotation yielded 626,833 (54.9% assigned to COGs) and 119,082 (61.96% assigned to COGs) protein-encoding genes for the pristine and contaminated metagenomes, respectively (**Supplementary Table S2C**). In addition, 186 and 51 assembled sequences of 16S rRNA genes were identified from the pristine and contaminated shotgun metagenomes (**Supplementary Table S2C**). The original FW106 metagenomic DNA was also resequenced using Illumina using the same strategy as described above (Hemme et al., 2010).

To complement the metagenomic sequencing, the V4 region of the 16S rDNA genes in each metagenome were sequenced with Illumina MiSeq. A total of 2,945 and 247 OTU’s were defined for the pristine and contaminated metagenomes, respectively (**Supplementary Table S3**). Comparisons between the 16S gene-based genera abundance profiles generated by the different sequencing methods (MiSeq V4 region, metagenome reads from HiSeq and GAIIX) were calculated by Pearson (correlation) and Bray–Curtis (dissimilarity) methods (**Supplementary Table S3**). Sequences for each environment showed similar taxonomic abundance profiles at the genus level with high correlations, regardless of method and/or sample (**Supplementary Table S3**), indicating that the 16S sequence results from these samples are quite robust. However, no significant correlation was observed when abundance was calculated for individual OTUs as opposed to genera, likely due to the random distribution of short reads along the 16S gene that complicates clustering of 16S data from shotgun metagenomes. Chao1 estimates of species richness of the two metagenomes were 7260 and 845 for FW301 and FW106, respectively (**Supplementary Table S4**). Alpha diversity and evenness indices show a more diverse and even community in the pristine site (**Supplementary Table S4**). Rarefaction curves for the 16S amplicons sequenced from the two communities show that the pristine library was highly diverse and did not reach saturation while the contaminated library reached saturation quickly (**Figure 1A**). Rank abundance plots of the same data confirmed that the pristine metagenome showed a shallow curve indicative of a diverse and evenly distributed community while the steep curve of the plot for the contaminated metagenome confirmed dominance by a few populations (**Figure 1B**).

Assignment of 16S-based OTU’s to microbial taxonomy showed that both communities were dominated by *Bacteria* with a low abundance of *Archaea* (∼3% pristine, >1% contaminated; **Figures 2A,B**; **Supplementary Figure S1A**). The pristine showed a high level of microbial diversity, with almost 20% of the diversity represented by *Pseudomonas* (10%)*, Burkholderia* (3%)*, Massilia* (2%)*, Acidovorax* (2%), and *Aquabacterium* (1%) and the remaining 81% of sequences cumulatively represented by less abundant populations (individually <1%; **Supplementary Figures S1A,B**). In contrast, the contaminated community was dominated by *Rhodanobacter* populations (82% total, 79% dominant OTU) with smaller populations of *Burkholderia*, *Herbaspirillum*, and *Leptobacterium* and only 6% of remaining OTU’s cumulatively representing low abundance populations (individually <1%; **Supplementary Figures S1A,B**). These results are consistent with phylogenetic profiling of protein-coding genes from the metagenome annotations. Of the 3,091 unique OTUs detected, 2644 were only identified in the pristine metagenome, and only 101 (3.3%) were shared between the two communities (**Supplementary Figure S1C**). These shared OTUs include the dominant *Rhodanobacter* populations of the contaminated community, albeit in extremely low abundance (<1%) in FW301 (three amplicons in pristine vs. 8,383 amplicons in contaminated). About 92% of the diversity of the pristine site was undetected (i.e., below the level of detection) in the contaminated community whereas 4.7% of the total OTUs were enriched. The 16S sequence results were consistent with phylogenetic distributions observed from direct metagenomic sequencing and suggested that pristine groundwater harbors highly diverse microbial communities that decline drastically upon anthropogenic perturbation.

### Comparative Metabolic Analyses of OR-IFRC Metagenomes

The metabolic diversity of the OR-IFRC samples was analyzed using IMG and MG-RAST analysis tools and COG abundance profiling using STAMP (Parks and Beiko, 2010) (**Figure 3**; **Supplementary Figure S2**; **Supplementary Tables S5**–**S6**). In general, the pristine site showed a high metabolic potential as indicated by the broad range of metabolisms spread across multiple lineages (**Figure 3**). Much of the presumed loss of metabolic diversity in FW106 was attributed to the loss of phylogenetic diversity in the metagenome, with *Rhodanobacter* populations and their associated metabolisms dominating the community.

### Carbon Cycling

Analysis suggests both communities were primarily heterotrophic, with the pristine community showing a broader range of complex carbohydrate metabolisms such as lignocellulose degradation, though neither metagenome showed a high abundance of *Clostridia* species or of genes encoding cellulosome components (**Table 1**). The FW301 community also possessed alternative central carbon metabolism pathways to glycolysis such as the Entner-Doudoroff pathway and the methylglyoxal shunt. While both communities appear capable of degrading xylan and metabolizing xylose, the pristine metagenome indicated metabolism of a wider variety of pentose sugars, sucrose, and carboxylic acids. The pristine community is thus expected to have a higher capacity for degrading complex carbohydrates than the stressed community.

**Table 1:** Phylogenetic profiling of OR-IFRC metagenomes with select organisms involved in geochemical cycling.

Organisms	Pristine (FW301)			Contaminated (FW106)		
		
	PhyPro^a^	16S^b^		PhyPro^a^	16S^b^	
				
		Count	%^c^		Count	%^c^
**Acetogenesis**						
*Clostridium carboxidivorans*	82/47/1	–	–	1/0/0	–	–
*Clostridium ljungdahlii*	70/32/0	–	–	17/0/0	–	–
**Bacterial methane oxidation**						
* Methylococcus capsulatus*	260/375/22	76	1.05	154/173/6	4	0.04
*Methylomonas*	–/–/–	1	0.01	–/–/–	0	0
*Methylosinus trichosporium*	–/–/–	1	0.01	–/–/–	0	0
*Methylosphaera*	–/–/–	2	0.03	–/–/–	0	0
*Methylobacter tundripaludum*	439/474/25	1	0.01	189/205/6	0	0
**Archaeal methane**						
**Oxidation/Methanogenesis**						
*Methanosarcina barkeri*	196/94/0	0	0	3/2/0	0	0
*Methanohalobium evestigatum*	93/42/3	6	0.08	1/0/0	0	0
**Denitrification**						
*Rhodanobacter* sp^d^	–/–/–^d^	13	0.18	–/–/–^d^	9041	85.33
*Paracoccus denitrifwans*	117/130/11	0	0	17/43/1	0	0
*Pseudomonas stutzeri*	167/302/46	795	L1.02	147/201/2	6	0.06
*Pseudomonas fluorescens*	995/7042/10181			337/440/17		
*Pseudomonas syringae*	552/1115/569			269/244/14		
**Nitrification**						
* Chromobacterium violaceum*	1016/5507/1555	2	0.01	127/95/4	0	0
*Methylococcus capsulatus*	260/357/22	76	1.05	154/173/6	3	0.04
*Nitrococcus oceani*	209/252/18	1	0.01	134/131/3	0	0
*Nitrosomonas eutropha*	115/185/11	0	0	104/170/6	0	0
*Nitrospira multiformis*	355/596/25	13	0.37	205/176/4	1	0.01
*Paracoccus denitrifwans*	117/130/11	0	0	17/43/1	0	0
*Nitrobacter winogradskyi*	104/239/41	5	0.07	15/11/0	0	0
**Anammox**						
* Candidatus Kuenenia stuttgartiensis*	939/599/10	0	0	27/22/0	0	0
**Nitrogen fixation**						
*Anabaena variabilis*	171/102/1	0	0	8/1/0	0	0
*Azotobacter vinelandii*	243/275/46	0	0	176/217/3	0	0
*Rhodobacter capsulatus*	51/44/6	1	0.01	2/7/0	0	0
*Bradyrhizobium japonicum*	764/1443/249	9	0.11	55/62/12	2	0.02
*Afipia* sp.	142/387/46	1	0.01	68/92/24	0	0
*Rhizobium etli*	304/402/29	3	0.04	43/51/0	2	0.02
*Sinorhizobium* sp.	219/273/10	0	0	56/19/0	0	0
**Iron reduction**						
* Shewanella oneidensis*	51/43/8	3	0.04	21/14/1	0	0
*Geobacter sulfurreducens*	543/506/46	36	0.28	31/15/0	0	0
**Iron oxidation**						
* Acidithiobacillus ferrooxidans*	166/197/3	0	0	143/194/2	0	0
**Sulfate reduction**						
* Desulfovibrio vulgaris*	261/207/3	0	0	8/27/0	0	0
*Archaeoglobus fulgidus*	109/70/0	0	0	13/0/0	0	0
*Desulfotomaculum reducens*	198/137/2	0	0	10/1/0	0	0
*Desulfosporosinus* sp.	–/–/–	2	0.03	–/–/–	0	0
**Oxidation of sulfur compounds**						
*Rhodanobacter* sp.	–/–/–	13	0.18	–/–/–	9041	85.33
*Acidithiobacillus ferrooxidans*	166/197/3	0	0	143/194/2	0	0
*Thiobacillus denitrifwans*	280/527/48	1	0.01	155/160/12	0	0
*Chlorobium limicola*	82/76/5	0	0	6/13/0	0	0
*Allochromatium vinosum*	231/255/9	0	0	213/236/10	0	0
*Beggiatoa sp.*	380/285/6	8	0.11	96/52/1	0	0
*Thiothrix nivea*	193/151/3	–	–	193/151/3	–	–
**Polyphosphate accumulation**						
*Rhodanobacter* sp.	–/–/–	13	0.18	–/–/–	9041	85.33
*Candidates Accumulibacter phosphatis*	872/1898/133	0	0	193/255/13	0	0
**Dissimilatory metal reduction**						
*Cupriavidus metallidurans*	452/1577/710	69	0.96	140/304/58	0	0
*Ralstonia eutropha*	450/2568/3656	3	0.04	138/236/9	0	0
*Ralstonia pickettii*	389/785/149	3	0.04	215/272/25	0	0
*Ralstonia solanacearum*	516/1249/303	3	0.04	209/314/22	0	0
*Anaeromyxobacter dehalogenans*	623/603/19	54	0.75	58/35/0	0	0
*Desulfovibrio vulgaris*	261/207/3	0	0	8/27/0	0	0
*Desulfotomaculum reducens*	198/137/2	0	0	10/1/0	0	0
*Shewanella oneidensis*	51/43/8	3	0.04	21/14/1	0	0
*Geobacter sulfurreducens*	543/506/46	36	0.28	31/15/0	0	0


*^a^Numbers derived from IMG Phylogenetic Profiling (successive 30/60/90% BlastP identity cutoff).*

*^b^16S gene amplicons sequenced by Illumina MiSeq as described in section “Materials and Methods” (genus level information only). 16S amplicons are only classified to the genus level and may not accurately reflect strain diversity in the sample. Values are not included for Clostridium species due to the taxonomic diversity and poor classification of Clostridium species.*

*^c^Abundance of amplicons is expressed as a percentage of reads assigned to a particular genus compared to all OTU’s for the particular sample (7216 for FW301, 10571 for FW106).*

*^d^When the IMG phylogenetic profile was constructed for these metagenomes, Rhodanobacter genomic sequences were not present in the database and thus no Rhodanobacter denitrification genes were identified despite the fact that these genes are known to be present in the metagenomes.*

Carbon fixation did not appear to be a significant activity in either metagenome based on sequence annotation (**Table 1**). Photosynthetic organisms were not present in significant numbers in the subsurface and as such carbon fixation by photosynthesis was not predicted to be a significant pathway in either metagenome. Acetogenic *Clostridia* species were present in very low abundance in both metagenomes, but acetogenesis does not appear to be a significant source of carbon fixation in either community (**Table 1**) and what carbon fixation activity exists likely occurs via reductive TCA or related cycles. The pristine community showed a greater capacity for carbon fixation than the contaminated community, but the low abundance of carbon fixation systems suggested that the pristine community was also primarily heterotrophic, with the major sources of carbon for the community originating from carbon compounds leached from soil or being introduced from down-welling surface waters.

Experimental analysis of shallow groundwater systems suggested carbon monoxide cycling resulting from increased carbon turnover under aerobic conditions may be an important means of carbon cycling in shallow groundwater systems (Chapelle and Bradley, 2007). Despite the lack of acetogenic bacteria, the pristine metagenome showed a high abundance of *cox* carbon monoxide dehydrogenase genes capable of oxidizing CO to CO_2_ in the presence of oxygen (**Figure 3A**). In contrast, the abundance of CODH genes was extremely low in the contaminated community, suggesting that the process of carbon turnover may be significantly altered in the stressed community and that the overall rate of carbon turnover may be lower compared to the pristine system.

Methanogens and methanotrophs were present in both metagenomes at very low abundance (**Table 1**). Neither sequence annotation identified genes for methane monooxygenase (*pmoA*; *mmoX*), methyl-H_4_MPT:Coenzyme M methyltransferase (*mtrA*). Thus, methane metabolism is not expected to represent a significant activity in either groundwater community.

Aromatic compounds are expected in natural environments, often resulting from the degradation of lignin compounds, and are also high-concentration contaminants at the OR-IFRC sites. The pristine sample contained a higher proportion and diversity of aromatic degradation genes than the contaminated sample. The complement of aromatic degradation genes in the pristine sample included pathways for degradation of toluene, xylene, and benzoate derivatives. In contrast, the most abundant aromatic degradation genes in the contaminated sample were of the acetone carboxylase/acetophenone carboxylase family (**Figure 3A**). The pristine sample generally showed a broader diversity and higher abundance of such genes and more complete degradation pathways, with chlorinated hydrocarbon degradation genes generally in higher abundance for the contaminated metagenome.

### Nitrogen Cycling

While the distribution and breadth of carbon metabolism genes suggests a higher overall rate of carbon cycling in the pristine site, the situation is less clear for nitrogen metabolism (**Figure 3B**; **Table 1**). As with carbon metabolism, the pristine groundwater metagenome showed a broader diversity of metabolic genes involved in nitrogen cycling compared to the contaminated site (**Figure 3B**). Evidence for nitrogen fixation, nitrification, denitrification and anaerobic ammonium oxidation was observed (**Table 1**). This is in contrast to the stressed site where denitrification activity dominates (Hemme et al., 2010) (**Figure 3B**; **Table 1**). Nitrifying bacteria (*Chromobacterium, Nitrosomonas, Nitrococcus*, etc.) and anammox (*Kuenenia*) were present in both samples based on protein homology, but the abundance was generally lower in the contaminated site and few 16S genes for these species were observed by direct sequencing (**Table 1**). Metagenome annotation revealed 10 *nifH* genes (COG1348), all from the pristine site. The pristine community thus encodes a diverse complement of nitrogen cycling pathways.

### Sulfur and Phosphorous Cycling

Overall sulfur cycling appeared more complete in the pristine community and included sulfate reduction and sulfur compound oxidation genes that were more abundant in the pristine community than in the contaminated community. Sulfate-reducing bacteria were present at both sites in low abundance (**Table 1**). High nitrate concentrations and low pH typically inhibit sulfate-reduction activity, but sulfide-dependent nitrate reduction may still be possible in the stressed environment. An *sqr* (sulfide-quinone reductase) gene mapping to *Rhodanobacter thiooxydans* was identified in the contaminated metagenome, suggesting a population of this species may exist which could confer on the community the ability to couple oxidation of reduced sulfur compounds to denitrification. However, it is not clear if the concentrations of reduced sulfur compounds would be significant in the contaminated community due to low rates of sulfate reduction. Both metagenomes also showed *ppk* (polyphosphate kinase) and *ppx* (polyphosphatase) genes, with some of the *ppx* genes in the contaminated sample mapping to *Rhodanobacter* species (**Table 1**).

### Energy Metabolism

The pristine community generally showed a wide variety of both cytochrome *c* (particularly those related to formate dehydrogenase) and hydrogenase (Ni,Fe hydrogenase) encoding genes at similar abundances as the contaminated community. The contaminated community in general contained a higher abundance of genes encoding cytochrome *c* family proteins, in particular COG2863 (cytochrome *c_553_*) which is predicted to be involved in dissimilatory metal reduction (Hemme et al., 2010), and COG2010 (cytochrome *c*, mono- and di-heme variants; **Figure 3**; **Supplementary Table S5**). The abundances of iron-only and NiFe hydrogenases are low in both samples but slightly lower in the contaminated community, while Ni,Fe-hydrogenase III and formate hydrogenlyase genes are slightly more abundant in the contaminated community (**Figure 3A**; **Supplementary Table S5**). Molecular hydrogen levels are expected to be low under nitrate-reducing conditions (Lovley and Goodwin, 1988) such as are present at the contaminated site which may explain the relatively low abundance of these genes in that metagenome. Both samples thus showed a diversity of genes encoding cytochrome and hydrogenase proteins with individual profiles that likely reflect responses to differing environmental stresses.

### Heavy Metal Metabolism

Despite not being exposed to elevated concentrations of heavy metals, all of the genes encoding metal resistance mechanisms previously identified as being in high abundance in the contaminated metagenome were present in the pristine metagenome in relatively high abundance (**Figure 3B**; **Supplementary Table S6A**). These genes included divalent cation eﬄux (*czc, cadA, corA, chrA, mgtE*) and mercuric resistance genes (**Figure 3B**; **Supplementary Table S6B**). Several antibiotic resistance genes were also identified in the metagenomes at varying abundance (**Supplementary Table S6B**). Both metagenomes encoded a large number of broad specificity cation/multidrug eﬄux/MFS antibiotic transporters, a group of genes that impart resistance to a wide variety of lipophilic and amphiphilic inhibitors and may serve as a general stress response mechanism. The contaminated metagenome generally showed a higher abundance of these systems compared to the pristine metagenome. Many of the heavy metal and antibiotic resistance genes identified in the pristine metagenome mapped to plasmid and/or viral sequences, suggesting a mobile metagenome capable of rapid adaptation to these stresses (**Supplementary Table S6C**).

## Discussion

With future efforts to map the biodiversity of contaminated sites at the OR-IFRC, metagenomes from pristine sites are critical to serve as proper controls. While past analyses of stressed OR-IFRC metagenomes revealed an overabundance of geochemical resistance genes, these comparisons were made to the database of isolate genomes even today contain a large percentage of pathogen species or species not relevant to the OR-IFRC environment. The FW301 metagenome provides the proper control of analyzing these sites in their proper geochemical and ecological contexts.

The pristine metagenome showed a diverse complement of metabolic pathways (**Figure 4**). The metabolic potential of this community is redundant in the sense that multiple similar pathways are spread over a wide phylogenetic breadth. This in turn would impart robustness on the community in the sense that a loss of a single population would be unlikely to result in the loss of its metabolic pathways. The pristine community would thus be expected to endure some degree of environmental stress without permanent perturbation of the community. This is in contrast to the stressed community where the low biodiversity and dominance by *Rhodanobacter* results in a community whose metabolic potential approximates that of *Rhodanobacter*. In this environment, the loss of a single population could have significant effects on the metabolic potential of the community. This may explain the relative lack of aromatic degradation pathways in the stressed community despite the presence of high concentrations of aromatics in the environment.

The metabolic diversity and redundancy of the pristine environment also suggests efficient nutrient cycling despite the lack of photosynthesis or significant nitrogen fixation potential. The diverse array of geochemical cycling mechanisms implies an ability to rapidly convert nutrients or other compounds to useful forms with little leakage of nutrients from the ecosystem. This is in contrast to the stressed ecosystem in which significant stores of carbon (in the form of CO or aromatics) or nitrogen (nitrate converted to N_2_ as a detoxification measure) are likely unutilized by the system.

It has been previously observed that contaminated sites at the OR-IFRC have a latent metabolic potential (North et al., 2004; Fields et al., 2006; Moreels et al., 2008; Van Nostrand et al., 2009, 2011). Following denitrification and biostimulation, for example, iron- and sulfate-reducing activity increases significantly (Fields et al., 2006). The metagenome of the pristine site suggests such latent potential exists in this community as well. Over 80% of the biodiversity in the site is from low-abundance populations, including the dominant *Rhodanobacter* population of the FW106 community. If *Rhodanobacter* as a minor but nascent member of the pristine community can bloom to dominance under the right conditions, it seems likely that many other populations in the pristine community have the same potential. This also suggests future experiments to determine if the rise to dominance of certain populations from the pristine community is a stochastic process or the result of habitat selection. The pristine metagenome can thus be used to test hypotheses regarding microbial community theory in contaminated sites.

The latent potential of the pristine community also manifests as a reservoir of geochemical resistance genes, particularly a higher than expected reservoir of heavy metal resistance genes despite the lack of exogenous heavy metal contamination at the site. Many of these genes, including mercuric reductase operon genes, are known to be laterally transferred within populations at OR-IFRC sites (Coombs and Barkay, 2004; Martinez et al., 2006). This reservoir of genes represents a potential source of laterally transferable genes available to the community in the event of sudden and chronic exposure to exogenous contamination. The capacity to rapidly adapt to exogenous contamination thus seems to be built into the pristine community.

In summary, the sampled OR-IFRC pristine groundwater community is highly diverse and contains a wide variety of metabolic networks distributed across multiple phylogenetic lineages. Furthermore, the pristine community is likely robust to moderate environmental stresses and encodes a latent ability to rapidly adapt to high stress conditions. Knowledge of the nature of geochemical cycling in contaminated environments will aid in refining strategies for bioremediation of contaminated groundwater reserves and provide a predictive understanding of the effects of environmental contaminants (e.g., heavy metals, organics, antibiotics, etc.) on groundwater ecosystems that will provide insight into restoration of diverse ecosystem services.

## Sequence Deposition

The assembled FW106 and FW301 metagenome sequences are available at the US-DOE Joint Genome Institute Integrated Microbial Genomes (IMG/m) resource under taxon ids 3300000178 and 300000184 and as NCBI BioProjects PRJNA39533 and PRJNA258175.

## Conflict of Interest Statement

The authors declare that the research was conducted in the absence of any commercial or financial relationships that could be construed as a potential conflict of interest.

## Footnotes

1 http://public.ornl.gov/orifc/sitenarrative.cfm

2 http://www.esd.ornl.gov/orifrc/

3 http://soap.genomics.org.cn/soapdenovo.html

4 http://sourceforge.net/apps/mediawiki/amos/index.php?title=Minimus2
