# Supplementary material for: Comparative metagenomics reveals impact of contaminants on groundwater microbiomes
Source: Front Microbiol. 2015 Oct 31;6:1205. doi: 10.3389/fmicb.2015.01205 (PMC4628106; doi:10.3389/fmicb.2015.01205)
Supplement: Supplementary file 1 [file Data_Sheet_1.DOCX]

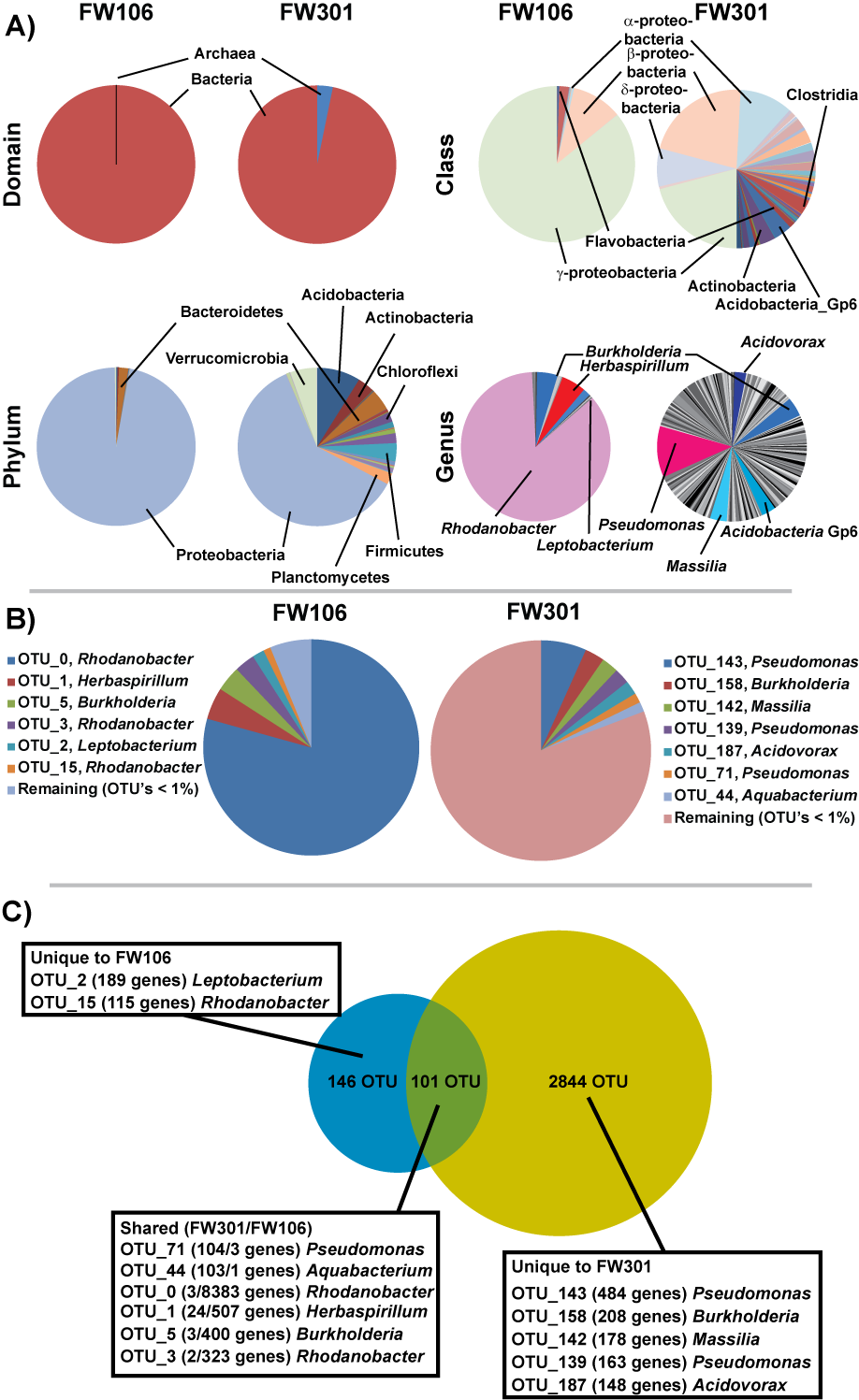


**Figure S1. OTU's of OR-IFRC metagenomes based on 16S amplicon sequencing.** A) Phylogenetic distribution of sequenced 16S V4 amplicons for OR-IFRC communities at the level of domain, phylum, class and genus. B) Phylogenetic identity of dominant OTU's in OR-IFRC metagenomes. "Remaining" category represents the sum of all OTU's with individual abundances <1%. C) Unique and shared OTU's between OR-IFRC metagenomes. OTU's with abundances >1% were labeled.
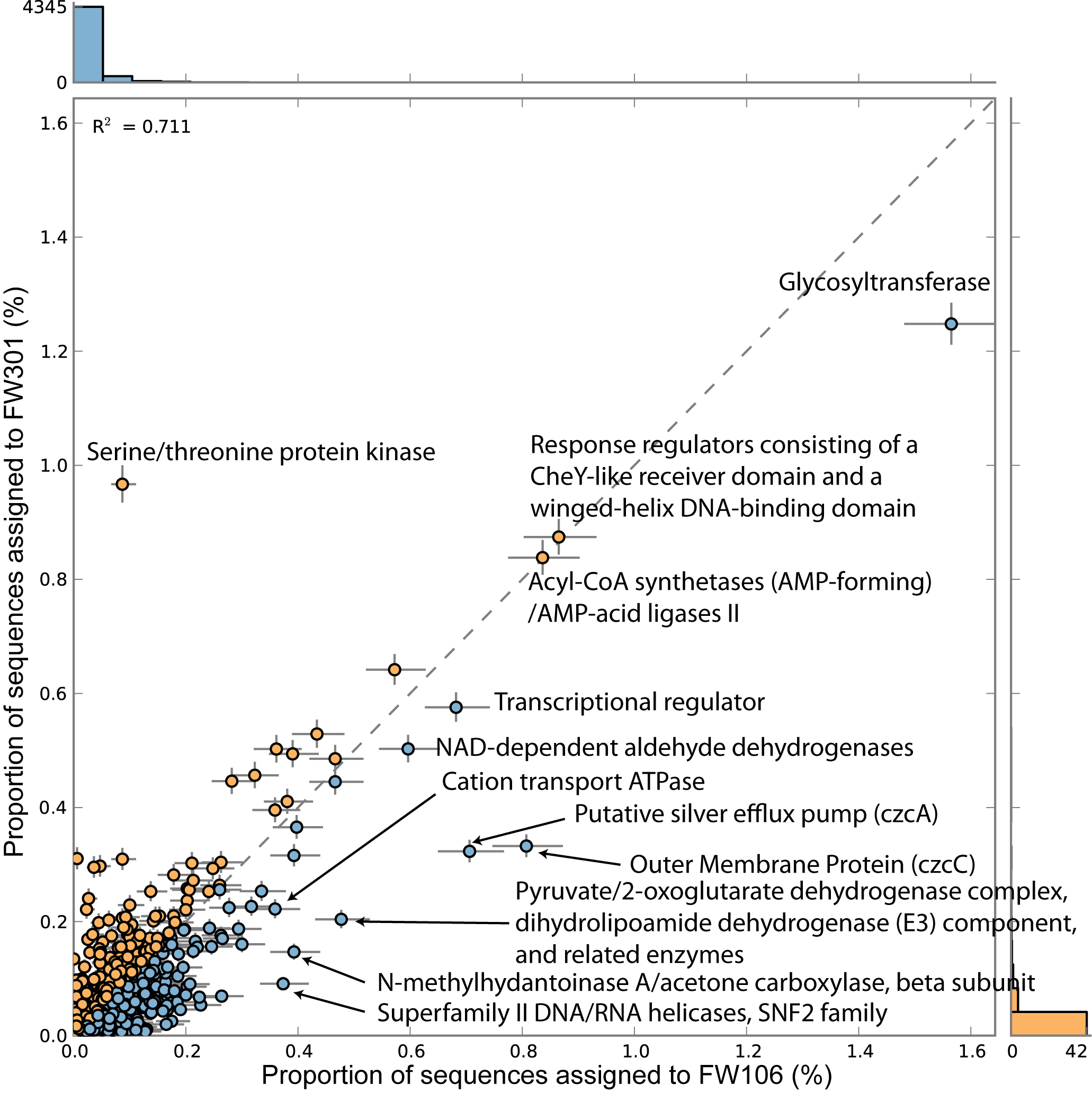


**Figure S2. Two-sample comparison of FW106 and FW301 metagenomes based on COG ID's.**  Samples are colored as orange (FW301, y-axis) and blue (FW106, x-axis). Plot was constructed in STAMP using the Two Sample Comparison tool (statistical parameters used listed in Materials and Methods). Axes measure proportions of sequences assigned to each feature.
