# Supplementary material for: Comparative metagenomics reveals impact of contaminants on groundwater microbiomes
Source: Front Microbiol. 2015 Oct 31;6:1205. doi: 10.3389/fmicb.2015.01205 (PMC4628106; doi:10.3389/fmicb.2015.01205)
Supplement: Supplementary file 2 [file Data_Sheet_2.DOCX]

Table S1. Geochemistry of the experimental sites FW301 (uncontaminated background) and FW106 (contaminated Area 3). Contaminant concentrations obtained from http://www.esd.ornl.gov/orifrc/ for the dates closest to the respective sampling periods.

|  | FW301 | FW106 |
| --- | --- | --- |
| pH | ~7 | 3.7 |
| NO_3_^-^ (mg/L) | 1.5 | 2331 |
| SO_4_^2-^ (mg/L) | 6.3 | 1997 |
| Uranium (mg/L) | >0.0001 | 51 |
| Technetium-99 (pCi/L) | - | 3700 |
| cis-1,2-Dichloroethene (μg/mL) | 5 | 1216 |
| 1,2-Dichloroethene (μg/mL) | 5 | 1153 |
| Tetrachloroethene (μg/mL) | 5 | 810 |
| 1-Butanol (μg/mL) | - | 475 |
| Acetone (μg/mL) | 10 | 823 |
| Benzoic Acid (μg/mL) | - | 1400 |
| Sodium (mg/L) | 1.96 | 826 |
| Chloride (mg/L) | 1.125 | 465 |
| Magnesium (mg/L) | 2.58 | 45.7 |
| Dissolved Oxygen (mg/L) | - | 0.26 |

**Table S2. Sequencing, scaffolding and annotation summaries of FW301 and FW106.** A) Sequencing statistics of Sanger and Illumina sequencing. B) Scaffolding statistics. C) Total number of RNA's and protein-coding genes resulting from IMG annotation. D) Annotation statistics for protein-coding genes.

A) *Sequencing Statistics*

| **Sample** | **Sequencing Technologies** | **Library size** | **(Average) read length** | **Number reads** | **Total size (Mb)** |
| --- | --- | --- | --- | --- | --- |
| FW301 | Sanger | 3,000 bp | 1,008 bp | 15,221 | 15.35 |
|  | Illumina GAIIx PE | 300 bp | 100 bp | 171,187,412 | 17,120 |
|  | Illumina HiSeq | 300 bp | 150 bp | 12,388,337 | 1,860 |
| FW106 | Sanger | 3,000 bp | 1,007 bp | 60,195 | 60.64 |
|  | Illumina GAIIx PE | 300 bp | 36 bp | 88,172,646 | 3,170 |
|  | Illumina HiSeq SE | 300 bp | 150 bp | 18,602,228 | 2,790 |

B) *Sequence Quality*

| **Sample** | **n** | **N:N50** | **Mean (bp)** | **N50 (bp)** | **Max (bp)** | **Sum (bp)** |
| --- | --- | --- | --- | --- | --- | --- |
| FW301 | 855,862 | 217,024 | 264 | 241 | 79,791 | 226.3e6 |
| FW106 | 76,169 | 17,498 | 769 | 720 | 280,116 | 58.58e6 |

C) *Sequence Annotation*

|  | **FW301** | | **FW106** | |
| --- | --- | --- | --- | --- |
|  | **Count** | **%** | **Count** | **%** |
| rRNA |  |  |  |  |
| Total | 2,872 | 100.00 | 724 | 100.00 |
| 16S | 186 | 6.47 | 51 | 7.04 |
| 18S | 27 | 0.94 | 6 | 0.82 |
| Protein Coding Genes |  |  |  |  |
| Total | 626,833 | 100.00 | 119,082 | 100.00 |
| With Product Name | 359,361 | 57.07 | 80,036 | 66.80 |
| With COG | 345,758 | 54.91 | 74,229 | 61.96 |
| With Pfam | 389,780 | 61.90 | 82,802 | 69.11 |
| With Kegg Orthology | 304,550 | 48.36 | 60,544 | 50.54 |
| With Enzyme | 177,800 | 28.24 | 34,352 | 28.67 |
| With KEGG Pathway | 191,404 | 30.40 | 35,698 | 29.80 |
| With MetaCyc | 124,204 | 19.72 | 24,295 | 20.28 |

**Table S3. Comparison of genera abundance of 16S samples in assigned OTU's based on different sequencing methods.** Correlation coefficients were determined in R using Pearson's method. Sequence fragments were taxonomically assigned using an internal IEG pipeline. Abundance was calculated by summing the sequence counts from all OTU's assigned to a given genus and dividing by the total number of sequences. "V4" indicates the amplicon was sequenced from the V4 region of the 16S gene, "Random" indicates a randomly located sequence read.

|  |  | FW301 | | | | FW106 | |
| --- | --- | --- | --- | --- | --- | --- | --- |
| Pearson |  | MiSeq | HiSeq | GAIIX  (Run 1) | GAIIX  (Run 2) | MiSeq | GAIIX |
| FW301 | MiSeq | 1.0000 | 0.8855 | 0.9003 | 0.8990 | - | - |
|  | HiSeq |  | 1.0000 | 0.9480 | 0.9483 | - | - |
|  | GAIIX (Run 1) |  |  | 1.0000 | 0.9990 | - | - |
|  | GAIIX (Run 2) |  |  |  | 1.0000 | - | - |
| FW106 | MiSeq |  |  |  |  | 1.0000 | 0.8331 |
|  | GAIIX |  |  |  |  |  | 1.0000 |
| Bray-Curtis |  | MiSeq | HiSeq | GAIIX  (Run 1) | GAIIX  (Run 2) | MiSeq | GAIIX |
| FW301 | MiSeq | 0.0000 | 0.6103 | 0.4768 | 0.4710 | - | - |
|  | HiSeq |  | 0.0000 | 0.6867 | 0.6810 | - | - |
|  | GAIIX (Run 1) |  |  | 0.0000 | 0.0366 | - | - |
|  | GAIIX (Run 2) |  |  |  | 0.0000 | - | - |
| FW106 | MiSeq |  |  |  |  | 0.0000 | 0.6471 |
|  | GAIIX |  |  |  |  |  | 0.0000 |
| Total Assembled Sequences | | 7,216 | 23261 | 4606 | 4730 | 10,571 | 5848 |
| No of OTUs^a^/Clusters^b^ | | 2945^a^ | 11020^b^ | 4218^b^ | 4412^b^ | 247^a^ | 1056^b^ |
| Read Location | | V4 | Random | Random | Random | V4 | Random |

^a^OTU's defined for MiSeq-derived amplicons by IEG internal pipeline as described in Materials and Methods

^b^Clusters were determined for 16S genes extracted from the read libraries using CD-Hit with 0.97 identity cutoff

**Table S4. Diversity Statistics.** Diversity statistics were calculated from the OTU abundance profiles using R using the *vegan* and *fossil* packages.

|  | **FW301** | **FW106** |
| --- | --- | --- |
| **Chao1** | 7260 | 845 |
| **Shannon** | 6.86 | 1.13 |
| **Simpson** | 0.99 | 0.37 |
| **Inverse Simpson** | 121.4 | 1.6 |
| **Pielou Evenness** | 0.859 | 0.205 |

**Table S5.** **Abundances of Cytochromes and Hydrogenase Genes Identified in the Metagenomes.** Abundances are expressed as percentages of the number of protein-coding genes assigned to a particular COG ID compared to all protein-coding genes assigned to all COG IDs (74229 and 345758 for FW106 and FW301, respectively).

| COG ID | Description | FW106 | | FW301 | |
| --- | --- | --- | --- | --- | --- |
|  |  | **Count** | **%** | **Count** | **%** |
| Cytochrome *c* |  |  |  |  |  |
| 1858 | Cytochrome *c* peroxidase | 20 | 0.027 | 157 | 0.045 |
| 2010 | Cytochrome *c*, mono- and diheme variants | 90 | 0.121 | 129 | 0.037 |
| 2857 | Cytochrome *c_1_* | 37 | 0.050 | 91 | 0.026 |
| 2863 | Cytochrome *c_553_* | 139 | 0.187 | 178 | 0.051 |
| 2993 | *cbb_3_*-type cytochrome oxidase, cytochrome *c* subunit | 24 | 0.032 | 70 | 0.020 |
| 3005 | Nitrate/TMAO reductases, membrane-bound tetraheme cytochrome *c* subunit | 0 | 0.000 | 18 | 0.005 |
| 3043 | Nitrate reductase cytochrome *c*-type subunit | 0 | 0.000 | 4 | 0.001 |
| 3245 | Cytochrome *c_5_* | 37 | 0.050 | 31 | 0.009 |
| 3258 | Cytochrome *c* | 37 | 0.050 | 91 | 0.026 |
| 3303 | Formate-dependent nitrite reductase, periplasmic cytochrome *c_552_* subunit | 1 | 0.001 | 17 | 0.005 |
| 3474 | Cytochrome *c_2_* | 7 | 0.009 | 43 | 0.012 |
| 3909 | Cytochrome *c_556_* | 14 | 0.019 | 13 | 0.004 |
| 4654 | Cytochrome *c_551_*/*c_552_* | 8 | 0.011 | 42 | 0.012 |
| Hydrogenase |  |  |  |  |  |
| 0374 | Ni,Fe-hydrogenase I large subunit | 0 | 0.000 | 55 | 0.016 |
| 0437 | Fe-S-cluster-containing hydrogenase components 1 | 3 | 0.004 | 171 | 0.049 |
| 1142 | Fe-S-cluster-containing hydrogenase components 2 | 15 | 0.020 | 3 | 0.001 |
| 1740 | Ni,Fe-hydrogenase I small subunit | 0 | 0.000 | 24 | 0.007 |
| 1969 | Ni,Fe-hydrogenase I cytochrome *b* subunit | 0 | 0.000 | 12 | 0.003 |
| 3260 | Ni,Fe-hydrogenase III small subunit | 17 | 0.023 | 46 | 0.013 |
| 3261 | Ni,Fe-hydrogenase III large subunit | 40 | 0.054 | 95 | 0.027 |
| 3262 | Ni,Fe-hydrogenase III component G | 5 | 0.007 | 12 | 0.003 |
| 4237 | Hydrogenase 4 membrane component (E) | 21 | 0.028 | 28 | 0.008 |
| 4624 | Iron only hydrogenase large subunit, C-terminal domain | 0 | 0.000 | 6 | 0.002 |

**Table S6. Distribution of key geochemical resistance genes in OR-IFRC metagenomes.** A) Metal resistance genes. B) Antibiotic resistance genes. Abundances are expressed as percentages of the number of protein-coding genes assigned to a particular COG ID compared to all protein-coding genes assigned to all COG IDs (74229 and 345758 for FW106 and FW301, respectively). C) Plasmid and viral genes in FW301 and FW106 metagenomes based on phylogenetic assignment of protein-coding genes by IMG (successive 30%/60%/90% percent identity). Domains: P (Plasmid), V (Virus)

A) *Metal Resistance Genes*

| **COG ID** | **Product** | **FW301** | | **FW106** | |
| --- | --- | --- | --- | --- | --- |
|  |  | **Count** | **%** | **Count** | **%** |
| 0598 | CorA | 185 | 0.054 | 31 | 0.042 |
| 2217 | CadA | 859 | 0.248 | 298 | 0.401 |
| 0672 | FTR1 | 38 | 0.011 | 58 | 0.078 |
| 0789 | ACR3 | 62 | 0.018 | 33 | 0.044 |
| 3696 | CzcA | 1,250 | 0.361 | 586 | 0.789 |
| 0841 | CzcA | 2,482 | 0.718 | 475 | 0.640 |
| 0845 | CzcB | 276 | 0.080 | 166 | 0.224 |
| 1538 | CzcC | 643 | 0.186 | 335 | 0.451 |
| 1230 | CzcD | 157 | 0.045 | 115 | 0.155 |
| 0861 | TerC | 138 | 0.040 | 24 | 0.032 |
| 1275 | TehA | 8 | 0.002 | 17 | 0.023 |
| 2059 | ChrA | 103 | 0.030 | 62 | 0.084 |
| 0474 | MgtA | 419 | 0.121 | 120 | 0.162 |
| 2239 | MgtE | 128 | 0.037 | 39 | 0.053 |

B) *Antibiotic Resistance Genes*

| **Description** | **Category** | **FW301** | | **FW106** | |
| --- | --- | --- | --- | --- | --- |
|  |  | **Count** | **%** | **Count** | **%** |
| β-lactamase Class A | COG2367 | 43 | 0.012 | 3 | 0.004 |
| β-lactamase Class C | COG1680 | 352 | 0.102 | 19 | 0.026 |
| β-lactamase Class D | COG2602 | 21 | 0.006 | 0 | 0.000 |
| Cation/multidrug efflux pump | COG0841 | 2,482 | 0.718 | 475 | 0.640 |
| Small multidrug resistance protein | Pfam00893 | 37 | 0.011 | 11 | 0.015 |
| Uncharacterized vancomycin resistance protein | COG2720 | 19 | 0.005 | 0 | 0.000 |

C) *Plasmid and Viral Genes*

| **Domain** | **Phylum/Class** | **FW301** | **FW106** |
| --- | --- | --- | --- |
| P | Euryarchaeota | 1/1/0 | 0/0/0 |
| P | Actinobacteria | 32/38/18 | 1/0/1 |
| P | Bacteroidetes | 2/2/0 | 0/0/0 |
| P | Bacilli | 10/6/0 | 4/0/0 |
| P | Nitrospirae | 4/1/0 | 0/0/0 |
| P | Alphaproteobacteria | 76/100/47 | 78/70/11 |
| P | Betaproteobacteria | 19/27/27 | 71/82/47 |
| P | Gammaproteobacteria | 125/203/76 | 110/81/24 |
| P | Unclassified | 22/41/5 | 70/142/4 |
| P | Thermi | 1/0/0 | 0/0/0 |
| P | Basidiomycota | 1/0/0 | 0/0/0 |
| P | Unclassified Eukaryota | 1/0/0 | 0/0/0 |
| V | Retro-Transcribing Viruses | 1/0/0 | 0/0/0 |
| V | dsDNA Viruses No RNA Stage | 579/167/8 | 0/0/0 |
